# Differences in the selection response of serially repeated color pattern characters: Standing variation, development, and evolution

**DOI:** 10.1186/1471-2148-8-94

**Published:** 2008-03-26

**Authors:** Cerisse E Allen, Patrícia Beldade, Bas J Zwaan, Paul M Brakefield

**Affiliations:** 1 Institute of Biology, Leiden University, PO Box 9516 2300 RA Leiden, The Netherlands

## Abstract

**Background:**

There is spectacular morphological diversity in nature but lineages typically display a limited range of phenotypes. Because developmental processes generate the phenotypic variation that fuels natural selection, they are a likely source of evolutionary biases, facilitating some changes and limiting others. Although shifts in developmental regulation are associated with morphological differences between taxa, it is unclear how underlying mechanisms affect the rate and direction of evolutionary change within populations under selection.

Here we focus on two ecologically relevant features of butterfly wing color patterns, eyespot size and color composition, which are similarly and strongly correlated across the serially repeated eyespots. Though these two characters show similar patterns of standing variation and covariation within a population, they differ in key features of their underlying development. We targeted pairs of eyespots with artificial selection for coordinated (concerted selection) versus independent (antagonistic selection) change in their color composition and size and compared evolutionary responses of the two color pattern characters.

**Results:**

The two characters respond to selection in strikingly different ways despite initially similar patterns of variation in all directions present in the starting population. Size (determined by local properties of a diffusing inductive signal) evolves flexibly in all selected directions. However, color composition (determined by a tissue-level response to the signal concentration gradient) evolves only in the direction of coordinated change. There was no independent evolutionary change in the color composition of two eyespots in response to antagonistic selection. Moreover, these differences in the directions of short-term evolutionary change in eyespot size and color composition within a single species are consistent with the observed wing pattern diversity in the genus.

**Conclusion:**

Both characters respond rapidly to selection for coordinated change, but there are striking differences in their response to selection for antagonistic, independent change across eyespots. While many additional factors may contribute to both short- and long-term evolutionary response, we argue that the compartmentalization of developmental processes can influence the diversification of serial repeats such as butterfly eyespots, even under strong selection.

## Background

Despite the diversity of animal form in nature, a limited range of phenotypes is often observed [1-3]. Stasis, convergence, and limits in the direction of morphological evolution occur within lineages even when strong selection is implicated in divergence [4-7]. The propensity for phenotypes to evolve, or evolvability [8-11], is determined by the capacity of genetic and developmental systems to produce heritable variation and the action of selection on those variants [12,13]. Consequently, rates of adaptive change and diversification are expected to depend critically on underlying developmental mechanisms [2,5,9,14-16]. Several different lines of evidence provide more understanding of the role development plays in determining the direction of evolutionary change, including the documented association between morphological differences and changes in regulation of developmental-genetic pathways (reviewed in [17]), analysis of the variational properties of different developmental mechanisms [18-20], and analyses of the distribution of morphological characters in a given phylogeny [21]. How development affects the rate and direction of evolutionary change in contemporary populations under selection, however, remains largely unexplored [22].

### Diversification of serial repeats

The elaboration and differentiation of serially repeated characters provides clear examples of the association between underlying mechanisms and morphological evolution. The complexity and diversity of arthropod body segments, tetrapod limbs, vertebrate teeth, and nymphalid butterfly wing color patterns all result from the evolutionary differentiation of homologous serial repeats [23-26]. Individual elements often covary strongly, reflecting the effects of shared developmental pathways and mechanisms coordinating their development [25]. This covariance can affect rates of evolutionary change [5,15,27], potentially limiting the independent evolution of serial repeats. As a consequence, development can bias evolution (sensu [28]), as certain morphological changes may be more readily achieved than others. However, recent empirical studies demonstrate that strong limits predicted by patterns of covariation do not always prevent the independent evolution of morphological traits under artificial selection [29-31]. One plausible explanation for the discrepancy between theoretical predictions and empirical results is the failure of current evolutionary genetic models to account for the details of development that determine how phenotypic variation is generated, and thereby affect the rate and direction of evolutionary change [32-34]. Artificial selection experiments are a powerful tool for exploring limits on the process of morphological evolution, particularly in the evolution of correlated characters [35-43]. Here we focus on two characters with similar patterns of covariation among a set of serial repeats. These characters, elements of the color pattern on butterfly wings, differ in key aspects of their development. We use artificial selection to explore effects of these differences on the rate and direction of evolutionary change in this complex morphological phenotype.

### Evolution of eyespot patterns on butterfly wings

Within *Bicyclus *and other closely related butterfly genera, wing pattern diversification was probably driven in part by a long history of sexual and natural selection acting on the eyespots [44,45], which are serially repeated along the wing margins. Each eyespot consists of concentric rings of black and gold color surrounding a white central spot [46]. Color composition (the relative dimensions of the gold and black rings) and size are two important functional characteristics of eyespots [47,48] that differ in critical aspects of their development. Properties of an inductive signal (e.g., signal concentration) produced by cells in the eyespot organizer, the central focus [49], largely determine eyespot size [46,50,51], whereas color composition is determined by the threshold response of surrounding tissue to the concentration gradient of the diffusing focal signal [52,53]. Each of these two aspects of induction (signaling and response) appears to contribute differently to phenotypic variation in the eyespot pattern [54] and may have very different consequences for color pattern evolution.

When selection targets either the color composition or size of a single eyespot, all eyespots evolve in concert, even those on different wing surfaces [51,53]. Despite differences in the underlying development of color composition and size, each character is strongly and positively correlated across serially repeated eyespots and their main axes of variation [28] in captive *B. anynana *populations lie in the direction of concerted changes in two or more eyespots. We used artificial selection to search for limits in this phenotype space, and selected for concerted (along the main axis of variation) and antagonistic (orthogonal to the main axis) change in the color composition or size of two eyespots. By applying similar selection to both characters and using a comparative analysis we test whether the characters differ in their propensity to evolve independently among serial repeats. Given initial similarities in patterns of standing variation for both characters, we argue in favor of the hypothesis that known differences in the developmental determination of eyespot color composition and eyespot size affect their ability to respond to antagonistic selection, and thus to evolve independently in a population under selection.

## Results and Discussion

### Differences in selection response between eyespot color composition and size

**Figure 1:**
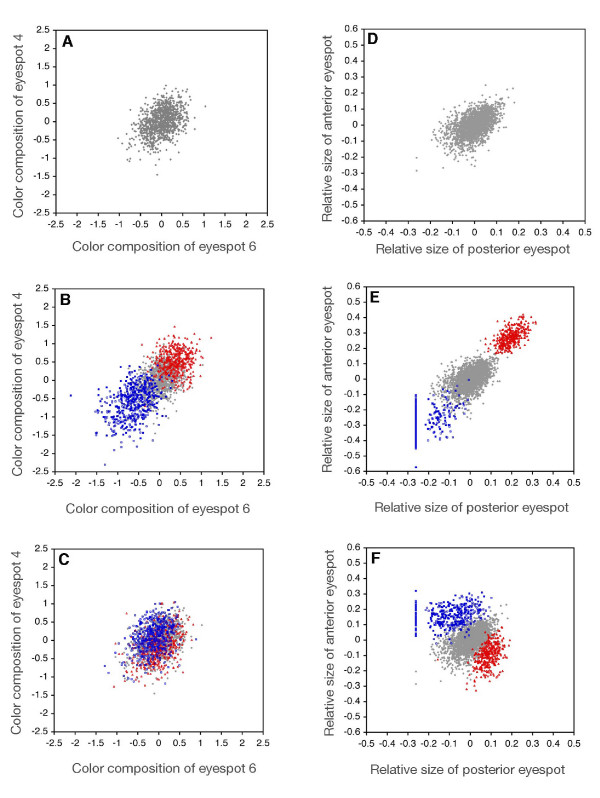
**Distribution of eyespot phenotypes before and after selection**. Bivariate phenotype distributions of eyespot color composition and size before and after artificial selection. **A-C**, color composition of ventral hindwing eyespots E4 and E6; **D-F**, size of dorsal forewing anterior and posterior eyespots. In all panels, grey points illustrate phenotype distributions in the starting populations at Generation 0. **B **and **E**, distribution of concerted selection lines at Generation 10 relative to Generation 0 (**B**: 'Black-Black' selected lines in red, 'Gold-Gold' selected lines in blue; **E**: 'Large-Large' lines in red, 'Small-Small' lines in blue). **C **and **F**, distribution of antagonistic selection lines at Generation 10 relative to Generation 0 (**C**: 'Black-Gold' in blue, 'Gold-Black' in red; **F**: 'Small-Large' in blue, 'Large-Small' in red). Filled and solid points denote replicates in each selection direction. All data for Generation 10 are shown relative to trait values at Generation 0 (see Materials and Methods for details of trait estimation and selection). Before selection, color composition and size are both positively correlated across pairs of eyespots, but each shows substantial variation in the direction corresponding to antagonistic selection (shown in Figure 2). For Generation 0, N = 1056 for color composition and N = 2254 for size; sample sizes for individual selection lines at Generation 10 ranged from N = 179 to N = 228 (color composition) and from N = 191 to N = 245 (size).

**Figure 2:**
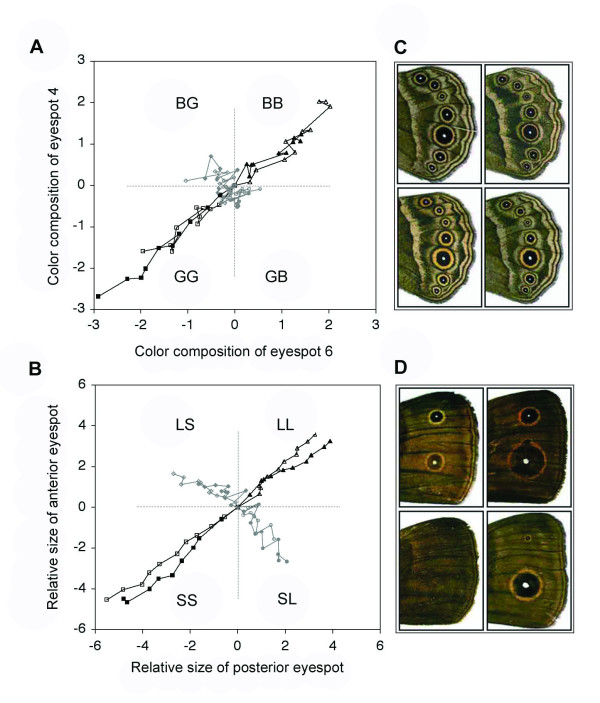
**Response to artificial selection for concerted and antagonistic changes in eyespot color composition and size**. **A **and **B**, Response each generation relative to unselected control values, plotted in phenotypic standard deviations from the starting population mean. Both characters were selected for concerted (black points and lines) and antagonistic (grey) change in two eyespots, filled and solid points represent replicate populations. Lines join points in consecutive generations and mean phenotypes for the starting populations are plotted at the origin. **A**, Selection for color composition of the fourth and sixth ventral hindwing eyespots (E4 and E6): 'BB' ('Black-Black') and 'GG' ('Gold-Gold') are concerted directions; 'BG' ('Black-Gold') and 'GB' ('Gold-Black') are antagonistic directions. **B**, Selection for size (relative to wing size) of the anterior and posterior eyespots on the dorsal forewing: 'LL' ('Large-Large') and 'SS' ('Small-Small') are concerted directions; 'LS' ('Large-Small') and 'SL' ('Small-Large') are antagonistic directions. **C **and **D**, Representative phenotypes for each selected direction in generation 10 (**C**, ventral hindwings shown for color composition lines; **D**, dorsal forewings shown for eyespot size lines; wings arranged according to axes in **A **and **B**).

Color composition and size were similarly correlated between the serially repeated eyespots in the *B. anynana *base population before selection (color composition: r_pearson _± SE = 0.42 ± 0.03, N = 1056, between ventral hindwing eyespots E4 and E6; size: r_pearson _± SE = 0.52 ± 0.02, N = 2254, between anterior and posterior dorsal forewing eyespots). For both characters, most phenotypic variation in the base population lay in the direction of positive covariation between two eyespots but each exhibited substantial variation around this main axis (Fig. 1a, d). Previous experiments demonstrated the genetic basis for these phenotypic correlations and the presence of substantial additive genetic variation for the size and color of individual eyespots [51,53,55,56]. As expected, both color composition and size responded rapidly to concerted selection imposed along the main axis of phenotypic variation (Fig. 1b, e; Fig. 2a, b). After 10 generations of concerted selection (see Materials and Methods), novel phenotypes well outside the range of variation in the base population were produced: color composition diverged ~2–3 SD (standard deviations, calculated from the base population) in the 'Black-Black' and 'Gold-Gold' directions relative to unselected control lines (Fig. 2a, c), and eyespot size diverged ~4–6 SD in the 'Large-Large' and 'Small-Small' directions relative to unselected controls (Fig. 2b, d). In contrast, responses to antagonistic selection demonstrate that the boundaries of phenotype space are very different for the two characters in this population (Fig. 1c, f; Fig. 2a, b). There is little potential for independent evolutionary change in eyespot color composition, compared with the strikingly flexible evolution of eyespot sizes.

After 10 generations of antagonistic selection, no novel 'uncoupled' eyespot phenotypes appeared in the 'Black-Gold' or 'Gold-Black' directions (Fig. 1c; Fig. 2a, c). The rate of response to antagonistic selection (scaled to the cumulative selection differential; Table 1) is clearly lower than the rate of response to selection for concerted changes in eyespot color composition. In some replicates there was no significant response to antagonistic selection for color composition (Table 1). Although selection can rapidly shift the mean color composition of the entire eyespot pattern (Fig. 1b; Fig. 2a, c), there are limits on the independent evolutionary change of individual eyespots. No comparable limits are apparent for eyespot size; antagonistic selection ('Large-Small' and 'Small-Large' directions; Fig. 1F, Fig. 2b, d) rapidly produced combinations of large and small eyespots that were not present in the starting population [57]. Moreover, the rate of response to antagonistic selection on eyespot size was as high (or higher, in some replicates) as the rate of response to concerted selection (Table 1).

**Table 1:** Rates of response to concerted and antagonistic selection.

Color composition			Size		
Line	Eyespot 4	Eyespot 6	Line	Anterior	Posterior
BB_1_	0.169 ^a^	0.146 ^d^	LL_1_	0.331 ^h^	0.308 ^n^
BB_2_	0.097 ^b^	0.095 ^e^	LL_2_	0.350 ^h^	0.301 ^n^
GG_1_	0.123 ^b^	0.110 ^e^	SS_1_	0.300 ^h, i^	0.370 ^l, m^
GG_2_	0.191 ^a^	0.161 ^d^	SS_2_	0.292 ^i^	0.358 ^m^
BG_1_	0.016^ NS^^ c^	0.034^f^	LS_1_	0.300 ^h, i^	0.437 ^k^
BG_2_	0.026† ^c^	0.032^f^	LS_2_	0.318 ^h, i^	0.427 ^k, l^
GB_1_	0.018^ NS^^ c^	0.017‡^f, g^	SL_1_	0.202 ^j^	0.264 ^n^
GB_2_	0.038 ^c^	0.002^ NS g^	SL_2_	0.290 ^i^	0.265 ^n^
ANCOVA	33.94**	26.33**		2.66*	10.04**

Values represent slopes from the regression of response to selection on cumulative selection differential, shown for each target eyespot in the color composition and size experiments. Rates of response were significantly different from zero with P < 0.001 except those marked by † (P < 0.005), ‡ (P < 0.01), and NS (P > 0.05). Nested analysis of covariance (ANCOVA) F-values are given for the interaction effect of cumulative selection differential by line nested within selection direction (df = 7,72); **P < 0.0001, *P < 0.01. Superscripts refer to slopes, for each eyespot within an experiment that were judged not significantly different from each other according to Tukey's pairwise multiple comparisons (α = 0.05).

### Potential causes of the difference in selection response

Our results show that under similar selection regimes, there are strong limits on the independent evolution of eyespot color composition that do not exist for eyespot size. This difference in evolutionary response occurred despite the fact that both characters exhibited substantial phenotypic variation in both concerted and antagonistic directions prior to selection (Fig. 1). Genetic correlations (measured in the outbred stock population; see Materials and Methods) across the targeted eyespots for color composition (r_G _± SE = 0.68 ± 0.15) and size (r_G _± SE = 0.58 ± 0.1) suggest that patterns of genetic covariation were also similar for both characters. The magnitude and direction of phenotypic and genetic correlations (and the relationship between the two; [58]) is generally taken to indicate the degree of limitation on the independent evolution of two or more characters [27,59-62]. Our experimental approach involved investigating the realized potential for independent evolution among repeats of two characters with similar patterns of standing variation known to differ in important aspects of their developmental determination. We expected to observe qualitatively similar responses of color composition and size in all selected directions if the patterns of covariation alone were the major internal factors governing the independent evolution of eyespot characters. While we argue that the degree of compartmentalization of the developmental mechanisms differs between color composition and size, we discuss additional factors that may also explain the observed evolutionary responses.

### Compartmentalization of focal signaling

Our results demonstrate much greater flexibility in the evolutionary response of eyespot sizes compared with color composition, despite the fact that both are characteristics of the same serially repeated color pattern elements on the wings of *B. anynana*. One explanation for this pattern is that the developmental pathways that regulate production of the focal signal (and determine eyespot size; [50,51,63]) are much more flexible and more easily decoupled across a wing surface than the pathways that regulate the threshold level of response to signal concentrations (and determine eyespot color composition; [52,53,64,65]). Each eyespot-competent area along the wing margin produces its own eyespot organizer [51,66]. This process appears to involve independent genetic control of organizer properties; single eyespots can be independently added to or eliminated from forewing or hindwing surfaces, without affecting the characteristics of eyespots in adjacent wing cells [67,68]. Eyespots are deleted from wing surfaces when focal establishment fails in specific wing cells [67,68], and eyespot-specific allelic effects are associated with changes in size of individual eyespots [66]. The wing veins act to further compartmentalize the signaling foci across a wing surface [45]. Compartmentalization (or modular organization; [10]) in the regulation of initially identical developmental programs is considered critical to the evolutionary divergence of serial repeats [69]. Selection acting on heritable, independent variation in developmental regulation can promote evolutionary diversification, even when similarities in underlying developmental programs contribute to positive covariation among the characters [70].

### Wing-wide responses to signal thresholds

**Figure 3:**
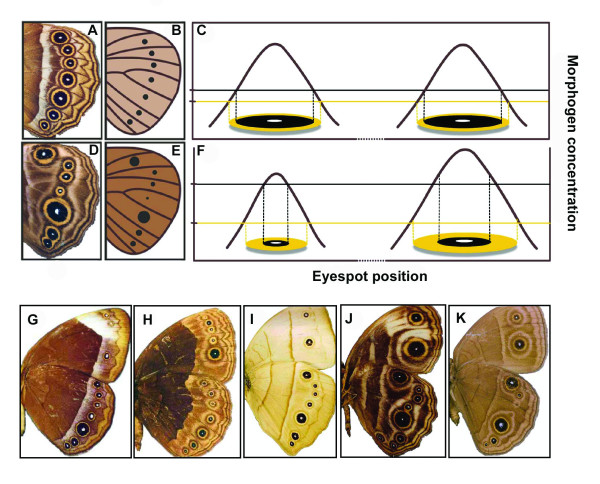
**Model for the evolutionary diversification of eyespot size and color composition**. **A **and **D**, phenotypes representing presumed 'ancestral' and 'derived' eyespot patterns, respectively. **B **and **E**, schematics illustrating the strength of the focal signal (size of the black dot) and the level of threshold response (shading of the wing background) for the two phenotypes. In **C**, eyespot foci at two positions on an 'ancestral' wing surface (x-axis) produce the same amount of a diffusing morphogen (brown curve). The threshold concentration of morphogen inducing black pigment formation (black horizontal line) is higher than the gold-inducing threshold (yellow horizontal line). Size and color composition are the same for both eyespots. In **F**, eyespot foci on a 'derived' wing surface produce different amounts of the morphogen signal (brown curves) and consequently differ in total size. When the threshold for black pigment production is increased, both eyespots are proportionately 'golder,' since threshold concentration is a property of the whole wing surface. **G-K**, *Bicyclus *wing patterns illustrating variation in eyespot color composition (across species, but not within a wing surface) and size (across species and individual eyespots). **G**, eyespots relatively black (*B. analis*); **H**, eyespots relatively gold (*B. buea*); **I-K**, clear individualization of eyespot size but not color composition within a wing surface (left to right: *B. italus*, *B. maesseni*, *B. milyas*).

**Figure 4:**
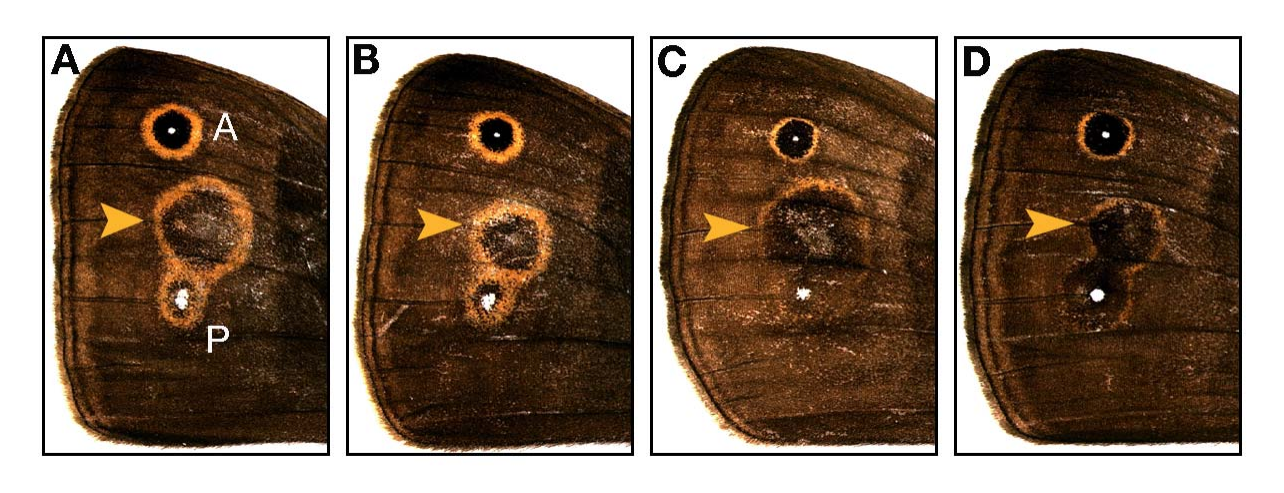
**Selection for eyespot color composition affects response properties of the wing epidermis**. Ectopic eyespots (arrows) were produced following damage to a non-focal position on the dorsal forewing 17 hours after pupation. Damage induces spatio-temporal patterns of gene expression in the surrounding tissue that mimic the response to diffusing focal signals [94] and results in an ectopic eyespot centered on the damaged site [46], located between the anterior (A) and posterior (P) eyespots. The color composition of ectopic eyespots was significantly different between 'GG' selected lines (**A **and **B**) and 'BB' selected lines (**C **and **D**; ANCOVA for effect of selection direction: F_2,138 _= 4.25, P < 0.01; Tukey's HSD: 'GG' vs. 'BB,' t_138 _= 6.7, P_adj _< 0.0001). The ectopic eyespots reveal evolutionary changes in the response properties of wing epidermal tissue after concerted selection for color composition. Earlier focal grafting and non-focal damage experiments [51, 53] demonstrate that selection for eyespot size mainly affects properties of the focal signal and the size of ectopic eyespots does not differ between lines selected for large and small eyespots [53, 95].

In contrast, the observed tight coupling of color composition across eyespots may result from a lack of compartmentalization in the regulation of threshold levels of response to the concentration gradient of the diffusing focal signal. Cells in the region surrounding the eyespot focus exhibit a threshold response to local signal concentration, which induces the expression of regulatory genes that control pigment synthesis (Fig. 3a–f; [45,52,53]). Earlier experiments clearly demonstrate that the signaling and response components of eyespot determination can be selected independently, and that selection for eyespot color composition affects only the level of threshold response [51,53]. In our experiment, concerted selection for eyespot color composition altered the response properties of the wing epidermis, even in regions of the wing that do not normally produce an eyespot and were not directly targeted by selection. The color composition of ectopic eyespots induced on wings of butterflies from concerted selection lines (Fig. 4) further suggest that the threshold response is not compartmentalized across comparable regions of a wing surface (spanning at least three wing cells) where there is clear compartmentalization of focal signaling [67,68]. If threshold response is regulated across regions spanning several wing cells (or across an entire wing surface [71]), variation in the color composition of two eyespots (Fig. 1a) might result from random perturbations during the development of individual wing compartments, not from independent heritable variation in response properties. Although the developmental-genetic architecture of the processes that regulate eyespot color composition requires further characterization, we suggest that the extent to which focal signaling and response thresholds are compartmentalized is a critical factor determining the potential for evolutionary change in the wing color pattern.

### Sources of covariation across wing surfaces

Selection targeted pairs of eyespots on two wing surfaces that differ in both morphology and ecological function. Eyespots on the dorsal [48] and ventral [47] wing surfaces are thought to function differently in *B. anynana*; however, all eyespots are determined by similar spatial and temporal patterns of gene expression [63,72] regardless of their location. This shared development appears to play a primary role in shaping covariation and strongly integrating eyespots among all wing surfaces in Nymphalid butterflies [44,73-76], although functional differentiation of some wing characters can also affect patterns of covariation [77]. Single- [51,53] and multi-trait selection on eyespot size [55] has demonstrated that the evolutionary potential of eyespots on all wing surfaces is strongly coupled and differs little among wing surfaces, despite differences in morphology or function. Antagonistic selection targeting the size of the two dorsal forewing eyespots also uncoupled individual eyespots on the ventral hindwing. The relative sizes of ventral hindwing eyespots E4 and E6 (the targets of selection for color composition) differed by ~2 SD in the antagonistic selection lines compared to unselected controls ([55]; ANOVA for effect of selection direction: F_4,495 _= 67.7, P < 0.0001; Tukey's HSD for relative size of eyespots 4 and 6 in 'Large-Small' vs 'Small-Large' lines: t_396 _= 8.3, P_adj _< 0.001). Thus the ability to decouple eyespot sizes by selection is clearly not limited to the dorsal forewing. In contrast, across a similar spatial region of the ventral hindwing (two eyespots separated by an intervening wing cell), eyespot color composition is strongly coupled and antagonistic selection cannot produce independent evolutionary changes.

### Alternative explanations

We suggest that known differences in the developmental determination of eyespot color composition and size contribute to qualitative differences in their responses to antagonistic selection. However, our experiments do not rule out the possibility that other factors contributed to these differences. In addition to developmental constraints or biases, factors that may limit the evolution of character combinations include the effects of past selection, the effects of inbreeding or genetic background, the action of maternal effects or gene-by-environment interactions, asymmetric gene frequencies or other differences in patterns of allelic effects, linkage disequilibrium or physical linkage, and correlation with other fitness-related characters (reviewed in [78,79]). Regardless of the precise mechanism, differences in selection response are unlikely to have been caused by differences in the two experimental populations. Both starting populations were derived from the same outbred stock population, which was well adapted to the laboratory and maintained at high effective population size [80] before selection began, and both experiments were conducted under similar environmental conditions. Consequently, neither differences in allele frequencies between the starting populations, nor in the interactions between genetic background and environmental conditions across experiments [81] are likely to explain the qualitative differences in selection response between the two characters.

Differences in the history (form and/or intensity) of natural selection acting on eyespot color composition and eyespot size is one possible source of differences between the characters that could influence their responses to antagonistic selection. Selection favoring specific character combinations can build or contribute to phenotypic and genetic covariation [30,36,37,82] via linkage disequilibrium. This type of covariation is expected to break down rapidly under antagonistic selection [83], and the breakdown of linkage disequilibrium created by past natural selection on eyespot size (for combinations of overall larger or smaller eyespots) may have contributed to the rapid independent evolution of eyespot sizes under antagonistic selection [57]. Differences in past selection acting on color composition and size provides one possible mechanism for differences in the underlying basis of covariation between the two characters. As comparable patterns of covariation may be reached through many different developmental or genetic mechanisms [84,85], this does not exclude our developmental hypothesis.

Our experiment explored whether limits exist on the evolution of particular types of eyespot combinations given standing variation in a population (Fig. 1a, d). Change in patterns of allelic interaction or allele frequency from those currently available in the population could affect the trait combinations or range of morphologies that can be reached via selection [34,79,84-86]. It is also possible that antagonistic selection acted on rare variants for eyespot size that, by chance, were not present for eyespot color composition in the stock population. Though our experiment does not rule out effects of several different underlying mechanisms, our results are consistent with theory suggesting that the mechanistic basis of character covariation is a more important predictor of evolutionary change than the correlation estimates alone [5,32,84,85]. Because developmental interactions both shape patterns of phenotypic and genetic covariation among characters and evolve in response to selection acting on those characters [9,87], genetic architecture, developmental mechanisms, and population-genetic processes interact in complex ways [88]. Understanding how these factors interact to shape the evolution of complex traits, such as butterfly wing patterns, requires further detailed dissection of underlying mechanisms.

### Diversification within the lineage

Phylogenetic patterns of morphological diversity are generally consistent with the idea that development limits the ability of species to occupy phenotype space (reviewed in [21]). In *B. anynana*, despite similar patterns of standing variation for eyespot color composition and size in the base population, responses to artificial antagonistic selection show that these two characteristics of the eyespot pattern do not evolve in the same way under similar antagonistic selection regimes. Moreover, these differences, found within a single *B. anynana *population, are consistent with wing pattern diversity across the genus (Fig. 3g–k; see also [89]). Among *Bicyclus *species, eyespot color composition seems to have evolved along a line of 'least resistance' [27,28] parallel to the main axis of variation in *B. anynana*. In contrast, eyespot size has explored a larger area of potential phenotype space along the 'uncoupling' axis [90].

## Conclusion

A number of studies suggest that natural selection plays a dominant role in morphological evolution and the distribution of lineages in phenotype space [29,30,36,39,57,91]. However, a growing body of evidence shows that developmental mechanisms and individual components of developmental gene networks can differ in their effects on phenotypic variation [19,20,54], and presumably, bias or limit adaptive morphological evolution. Here we compared two characters that show similar patterns of standing variation in a population, but differ in an important aspect of their developmental determination. These characters evolve differently under artificial antagonistic selection, in a manner consistent with the developmental differences between them, though we cannot rule out the effects of other internal factors or processes besides, or in addition to, development. Butterfly color patterns and other complex forms we see in nature result from novel combinations of their individual elements. We suggest that compartmentalized development enables the production of novel phenotypes when selection favors independent changes in these elements. Under similar selection, developmental mechanisms that are less compartmentalized may limit the range of likely evolutionary outcomes.

## Methods

### Selected traits

In *Bicyclus anynana*, two eyespots are present on the dorsal forewing (anterior, A, and posterior, P) and seven on the ventral hindwing (eyespots E1–E7). In separate experiments, we selected for the relative size (the ratio of total eyespot diameter divided by the distance between the centers of A and P; [57]) or the color composition (the size of the black color ring relative to total eyespot diameter) of two eyespots simultaneously. Selection for eyespot size targeted the two dorsal forewing eyespots [57]. The results of size selection showed that selection to uncouple A and P also uncoupled the relative sizes of anterior and posterior groups of ventral hindwing eyespots, and that the division between the two groups of ventral hindwing eyespots lay between E4 and E6 [55]. Thus, we chose to select on the color composition of E4 and E6 because: 1) the size experiment showed that these two eyespots could be decoupled, even when they were targeted only indirectly by selection (see Results and Discussion); and 2) color composition is a function of total eyespot diameter, so it was necessary to choose two eyespots of initially similar size and color composition before selection.

The starting population for each experiment was derived from an outbred stock maintained in the laboratory for > 100 generations at high N_e _[80]. For each trait we established three types of lines from the stock population: 1) antagonistic selection lines where two eyespots were selected in opposite direction (large A and small P, 'Large-Small,' or the opposite combination 'Small-Large'; E4 gold and E6 black, 'Gold-Black,' or the opposite combination 'Black-Gold'); 2) concerted selection lines where two eyespots were selected in the same direction ('Gold-Gold,' or 'Black-Black'; 'Large-Large,' or 'Small-Small'); and 3) unselected control (UC) lines.

To estimate eyespot color composition, total diameter and the diameter of the inner black disc of E4 and E6 were measured in the proximal-distal axis, along the midline of each eyespot (parallel to the wing veins). Color composition of an eyespot was estimated from the orthogonal (Type II) regression of black disc diameter on total eyespot diameter. In this analysis, positive and negative residuals indicate relatively black and relatively gold eyespots, respectively. To identify individuals for selection, we performed an additional orthogonal regression of E4 color composition on E6 color composition (from the previous regression). The regression line is similar to the axis of the first principal component. For concerted selection, we ranked the residuals parallel to the regression line (similar to individual loadings on the first principal component); extreme positive or negative residuals indicated that both eyespots were relatively black or relatively gold, respectively. For antagonistic selection, we ranked the residuals perpendicular to the regression line (similar to individual loadings on the second, orthogonal, principal component axis); extreme positive residuals indicated individuals where E4 was relatively black compared to E6, and extreme negative residuals indicated individuals where E6 was relatively black compared to E4. Selection for eyespot size was made on the basis of the additive combination of the ranks for each individual eyespot as explained in [44]. There were two replicates of each selection and each UC line, with one additional UC replicate in the size experiment. To estimate correlations between eyespot characters in the base population, we calculated Pearson product-moment correlations between the target eyespots in the starting population for each experiment.

### Selection procedure

Female butterflies were selected for 10 generations. For the color composition experiment, 1056 females were measured and randomly split into two groups and 40 females were randomly drawn from each group to establish the UC lines. Within each group, the remaining females were ranked and the 40 females with the most extreme phenotypes in each direction were selected, so that each selection line was replicated twice in total. For the size experiment, 2254 females were measured and randomly split into two groups after 40 females were drawn to establish the first UC line [44]. All selected females were placed with ~50 randomly chosen males and allowed to lay eggs. Rearing conditions for the stock population and selected lines have been described previously [51]. We maintained similar selection intensities in both experiments for generations 1–10: each line, per generation, we measured 140–240 females for color composition (mean ± SE: 209 ± 5) and 15–200 females for size (mean ± SE: 173 ± 3). We selected 40 females per line every generation in the color experiment; in the size experiment the number decreased to 35 females between generations 5–10 [44].

### Selection response

Each generation, trait values for each replicate line were calculated relative to their respective UC lines. A least-squares regression of selection response on cumulative selection differential was used to estimate the rate of response to selection for each replicate. We used nested analysis of covariance (ANCOVA) to test for differences in the rate of response between selection directions, with 'cumulative selection differential' as the covariate and 'replicate within direction' as a random effect. Tukey's multiple comparisons were used to compare responses of individual replicates when there was a significant interaction between the two main factors in the nested ANCOVA. Analyses were conducted using PROC GLM in SAS version 6.12 (SAS Institute, 1996).

### Induction of ectopic eyespots

Damage to pupal wings in the first 18 hours after pupation induces formation of ectopic eyespots on the dorsal forewing [46] and can be used to assay the threshold response of wing tissue to eyespot-inducing signals [46,53]. To determine the effect of selection for eyespot color composition on this threshold response, we damaged the pupal wings of females in each of the Black-Black, Gold-Gold, and UC lines and measured the color composition of the resulting ectopic eyespots. The experiment was conducted during generation 14 (lines were maintained under random mating conditions after generation 10, with 40 females and 55 males selected at random and ~250 offspring reared per line per generation).

To reduce the size of native eyespots (A and P) on the dorsal forewing and increase visibility of the induced ectopic, we first pierced the foci of the anterior and posterior eyespots on the left dorsal forewing of each individual 4.5 hours after pupation with a finely sharpened tungsten needle [46]. We then induced ectopic eyespots by piercing each of the left forewings at a site in the fourth wing cell (immediately distal to the normal location of the eyespots) 17 hours after pupation. We returned operated pupae to 27°C, froze newly emerged adults (after their wings had fully hardened), and measured the total diameter and inner black diameter of ectopic eyespots and the interpupil distance between the reduced A and P eyespots on each manipulated (left) wing. We also measured the total diameter and black disc diameter of P on the right dorsal forewing, and the interpupil distance on this unmanipulated (right) wing.

We used ANCOVA to test for differences in color composition of ectopic eyespots among Black-Black, Gold-Gold, and UC lines. With 'total ectopic diameter' as the covariate, we examined the fixed effect of 'selection direction (control, black, or gold)' and the random effect of 'replicate line nested within direction' on the size of the ectopic black disc. None of the interaction terms (selection direction by covariate and random effect by covariate) were significant and we used Tukey's HSD to test for differences in the adjusted means between Black-Black, Gold-Gold, and UC lines.

### Estimates of genetic covariation in the stock population

We used a paternal half-sib breeding design [92] to estimate quantitative genetic parameters in our outbred stock population. We randomly selected 100 virgin males from the stock population at adult eclosion; each male was mated sequentially to 2 virgin females. At hatching, ~30 eggs per female were transferred to mesh rearing cages and fed on young maize plants ad libitum until pupation. Full-sib offspring were reared together but densities were kept low to minimize interaction and competition between individuals. Rearing cages were moved every 4 days to randomize environmental effects within the growth chamber. Emerging adult offspring were allowed several hours for their wings to expand and fully harden before being frozen for later analysis.

Five female offspring were randomly selected from each of 174 full-sib families (representing 87 sires who successfully produced offspring by two dams each) and dorsal forewing eyespots A and P, and ventral hindwing eyespots E4 and E6 were measured as described above. We used our nested breeding design (full-sib dam families nested within sire families) to estimate sire, dam, and progeny variance and covariance components in SAS 6.12. Genetic correlations between eyespot pairs (and their standard errors) were calculated according to [93]; to eliminate potential inflation of the estimates by maternal effects, genetic correlations were calculated from the among-sire variance components only [92].

## Authors' contributions

CEA participated in the design of the study, carried out selection and wing damage experiments, performed the statistical analysis and drafted the manuscript. PB participated in the design of the study and carried out selection experiments. BJZ participated in the design and coordination of the study. PMB conceived of the study and participated in its design and coordination. All authors helped to draft the manuscript and all approved the final version.
